# Stereotactic radiosurgery alone for small cell lung cancer: a neurocognitive benefit?

**DOI:** 10.1186/1748-717X-9-218

**Published:** 2014-09-30

**Authors:** Eric Ojerholm, Michelle Alonso-Basanta, Charles B Simone

**Affiliations:** Department of Radiation Oncology, University of Pennsylvania, 3400 Civic Center Boulevard, PCAM-2 West, Philadelphia, PA 19104 USA

**Keywords:** Small cell lung cancer, Stereotactic radiosurgery, Whole-brain radiation therapy, Neurocognitive, Prophylactic cranial irradiation

## Abstract

Yomo and Hayashi reported results of stereotactic radiosurgery alone for brain metastases from small cell lung cancer. This strategy aims to avoid the neurocognitive effects of whole-brain radiation therapy. However, radiosurgery alone increases the risk of distant intracranial relapse, which can independently worsen cognition. This concern is heightened in histologies like small cell with high predilection for intracranial spread. The majority of study patients developed new brain disease, suggesting radiosurgery alone may not be an optimal strategy for preserving neurocognitive function in this population. We suggest whole-brain radiation therapy should remain the standard of care for small cell lung cancer.

## Correspondence/Findings

Letter to the Editor:

Yomo and Hayashi recently reported in *Radiation Oncology* their experience with upfront stereotactic radiosurgery (SRS) for brain metastases from small cell lung cancer (SCLC) [1]. The authors should be commended for this novel investigation. We agree that SRS might play a role in SCLC, particularly for treating a limited number of brain metastases after prior prophylactic cranial irradiation or prior whole-brain radiation therapy (WBRT). However, we echo the authors’ call for caution in adopting SRS alone as the initial approach for intracranial disease.

The strategy of upfront SRS is gaining increasing prominence [2], spurred by the excellent local control achieved with radiosurgery and by concerns about the side effects of WBRT. These concerns are bolstered by studies showing declines in neurocognition and quality of life in patients receiving WBRT [3–5]. On the other hand, increased intracranial tumor burden often drives cognitive dysfunction [6, 7], and a recognized downside of SRS alone is high rates of distant brain relapse. This concern is heightened in SCLC, a biologically aggressive tumor which disseminates to the central nervous system in approximately two-thirds of patients during the course of their disease [8]. Yomo and Hayashi report that 20 of 41 patients (49%) developed new brain metastases after initial SRS, and because follow-up imaging was available in only 34 cases, this rate could be interpreted as 20 of 34 (59%). The overall outcomes from this study suggest that radiosurgery may be a reasonable modality in well-selected patients with SCLC. However, it is also possible that in populations with high rates of intracranial relapse, an SRS approach might actually hinder – rather than help – cognitive function.

Which is more important for preserving neurocognition: limiting the volume of irradiated brain or maximizing intracranial tumor control? The answer is uncertain, and ongoing studies aim to resolve this question (e.g. NAGKC 12–01 testing WBRT versus SRS alone for five or more brain metastases and NCCTG N107C testing WBRT versus SRS following surgical resection of a brain metastasis). Should these trials favor SRS, we anticipate increased enthusiasm for radiosurgery in all tumor histologies – including SCLC – and Yomo and Hayashi’s study will be an important initial report in this population. In the meantime, however, we suggest that WBRT remain the standard of care for patients with brain metastases from SCLC.

## Author information

MAB is Chief of the Central Nervous System Service and CBS 2nd is Chief of the Thoracic Service in the Department of Radiation Oncology.

## Abbreviations

SRS: Stereotactic radiosurgery
SCLC: Small-cell lung cancer
WBRT: Whole-brain radiation therapy.
